# Management of Dyslipidemia in Secondary Prevention of Cardiovascular Disease: The Gap between Theory and Practice

**DOI:** 10.3390/jcm11154608

**Published:** 2022-08-08

**Authors:** Gloria Santangelo, Silvia Moscardelli, Pasquale Simone Simeoli, Marco Guazzi, Pompilio Faggiano

**Affiliations:** 1 Division of Cardiology, Department of Health Sciences, San Paolo Hospital, University of Milan, 20122 Milan, Italy; 2 Cardiothoracic Department Unit, Fondazione Poliambulanza, 25100 Brescia, Italy

Hypercholesterolemia is one of the most important modifiable risk factors for cardiovascular events (CV) representing the principal driving force in the development of atherosclerotic cardiovascular diseases (ASCVD) [1]. A large amount of evidence from genetic, epidemiologic and clinical intervention studies has firmly established that high serum levels of Low-Density Lipoprotein Cholesterol (LDL-C) cause the formation and growth of atherosclerotic plaque [1]. Thus, the 2019 European Society of Cardiology (ESC) Guidelines introduced more strict LDL-C targets than in the past, especially in secondary prevention for patients at very-high risk (LDL-C reduction of ≥50% from baseline and an LDL-C goal of <1.4 mmol/L or <55 mg/dL -Class I Level A) and for patients who experience a second vascular event within 2 years (LDL-C goal of <1.0 mmol/L or <40 mg/dL- Class IIb Level B) [2].

Old therapies, such as niacin, bile acid sequestrants and fibrates, have been shown to lower LDL-C but nowadays are not part of the treatment of hypercholesterolemia, due to their limited effect in reducing LDL-C [3], side effects [4] and due to a lack of evidence regarding the reduction of CV events in randomized controlled trials [5,6].

For many decades, statins (3-hydroxy-3-methylglutaryl-coenzyme A-HMG CoA-reductase inhibitors) have been the only hypolipidemic drugs available with a proven CV mortality and morbility benefit. At present, statins are the first line therapy, in ASCVD patients and in primary prevention subjects. Nonetheless, the main side effects, such as myalgias and the increase of blood liver enzymes, remain major limiting factors to therapeutic adherence. Approximately 50% of patients discontinue statin therapy within 1 year, and adherence decreases over time [7]. In addition, despite a maximally tolerated dose, 40% of patients do not reach the target LDL-C levels [4]. In the DA VINCI study [8], only 54% of patients were treated with the maximally tolerated dose of statins and achieved their risk-based LDL-C goal, according to the 2016 European Guidelines. Furthermore, the ESC recommendation changes between 2016 and 2019 have highlighted the difficulty in reaching the LDL-C targets in patients with high or very-high CV, despite the optimal lowering lipid therapy with statins [9,10].

With these premises, the current American and European Guidelines recommend a step approach, including the addition of ezetimibe and proprotein convertase subtilin-kexin type 9 inhibitors (PCSK9i), to maximally tolerated statin therapy, in cases where LDL-C levels remain not at the goal suggested [2]. The introduction of ezetimibe along with statin therapy has been shown to promote an additional lowering of 13–20% in LDL-C levels [11]. The ACTE study (Safety and Efficacy of Ezetimibe Added on to Rosuvastatin 5 or 10 mg Versus Up-Titration of Rosuvastatin in Patients with Hypercholesterolemia) demonstrated that doubling the dose of statin is less effective than combining with ezetimibe in order to significantly reduce LDL-C levels (5.7% vs. 21% reduction) [12]. Alirocumab and evolocumab, two monoclonal antibodies targeting PCSK9, a serine protease responsible for extracellular binding and subsequent degradation of the LDL receptor, showed a mean reduction of LDL-C by 43% to 64%, compared to standard treatment, in several trials. In patients at very-high risk not achieving their goal on a maximum tolerated dose of statin and ezetimibe, a combination with PCSK9i is recommended (as Class IIb Level C for primary prevention and Class I Level A for secondary prevention [2]. PCSK9 plays a role in atherogenesis through its expression on the crucial cells participating in atherosclerosis development (endothelial cells, vascular smooth muscle cells and macrophages), thereby its inhibition has multiple effects, including the stabilization of atherosclerotic plaque, antiplatelet effects, antineoplastic effects and anti-bacterial effects [13]. PCSK9i incrementally reduces LDL-C levels and the risk of major adverse CV events (MACE) when administered alongside high-intensity statin and/or ezetimibe. PCSK9i administration to coronary artery disease (CAD) patients with statin pre-treatment reduced LDL-C levels by ≥60% with concomitant reduction in CV events. This finding suggests that the incremental lipid-lowering therapy using PCSK9i might be beneficial for plaque volume stabilization and even regression in patients with prior high-intensity use of statin. It is known that statins produce favorable changes in plaque morphology and composition by reducing lipid content, attenuating inflammation and increasing fibrous cap thickness (FCT). These changes make plaque rupture, and the consequent myocardial infarction, less probable [14].

Two recent trials, in a chronic and acute setting, the ALTAIR [15] Trial and PACMAN AMI [16] Trial, have demonstrated that the addition of PCSK9i is associated with a significantly greater increase in FCT measured with optical coherence tomography and diminution in maximum lipid arc, compared with standard lipid-lowering therapy. Although the mechanisms remain uncertain and further studies with a larger sample size are needed, it is likely that plaque stabilization is due mainly to the marked reduction in LDL-C levels rather than to the potential anti-inflammatory effect of the PCSK9i. In a study by Ota et al. [17] including CAD patients, the lipid component of non-culprit coronary plaques, evaluated by near-infrared spectroscopy-intravascular ultrasound, was significantly decreased by PCSK9i.

Thus, the effects of statin combined with PCSK9i might be attributed to the stabilization and regression of residual vulnerable coronary plaques in patients with CAD [17]. Based on the current recommended guidelines, PCSK9i administration is likely to be considered for patients with a very high CAD risk, so its administration may contribute to secondary prevention in patients with non-culprit segment plaques regardless of plaque vulnerability [18,19]. Even after combining statins, ezetimibe and PCSK9i, some patient subgroups fail to achieve LDL-C targets requiring alternative treatments. Bempedoic Acid (BA) and Inclisiran are two promising molecules which can be used for hypercholesterolemia reduction to the recommended target. The BA works in the same biosynthetic pathway as the enzyme HMG CoA reductase, but it lies upstream along the path. BA, through the inhibition of adenosine triphosphate citrate lyase (ACLY), that converts citrate to acetyl CoA, up-regulates LDL receptors and lowers LDL-C levels. BA affects the target enzyme only in the cells that can mediate its conversion from the inactive to active form, so thanks to its liver-specific mechanism of action, BA showed no muscle-related adverse effects. In addition to its cholesterol-lowering potential, BA possesses strong systemic anti-inflammatory effects leading to significant ASCVD risk reduction [20]. Focusing on this patient-centric approach, the recently approved BA-ezetimibe combination could be tried prior to attempting PCSK9 inhibitors, which have certain limitations including injectable preparation and higher costs. The fixed drug combination of BA and ezetimibe has shown LDL-C reduction comparable to PCSK9i in clinical trials. In February 2020, thanks to the results of Cholesterol Lowering via Bempedoic Acid and ACL-inhibiting Regimen (CLEAR) trial, the US Food and Drug Administration approved once-daily oral BA in heterozygous familial hypercholesterolaemia or established ASCVD patients who need further LDL-C lowering in addition to dietary modifications and maximally tolerated statins. However, one major limitation of use of BA includes the lack of CV disease outcome data, which will be addressed in an ongoing clinical trial [20].

Inclisiran is a disruptive, first-in-class small interfering RNA (siRNA) that prevents the intracellular translation of PCSK9 messenger RNAs (mRNAs) to protein, through the creation of effector RNA-inducing silencing complexes (RISCs). The inhibition of PCSK9 synthesis up-regulates the number of LDL receptors on the hepatocytes, thus lowering the plasma LDL-C concentration. Inclisiran decreases the LDL-C levels by over 50% with one dose every 6 months, with a dose-dependent duration effect. Due to the twice-yearly injection, inclisiran could improve therapeutic adherence; therefore, it represents a valid alternative to PCSK9i. While PCSK9i acts at the plasma level, inclisiran has an impact on the intracellular level (hepatocytes), without the involvement of the PCSK9 protein in the degradation of LDL receptors in lysosomes, which facilities liver regeneration and decreases the risk of hepatic damage. Furthermore, a cost-effectiveness analysis, adjusted for Australian healthcare, displayed that the price of inclisiran would have to be 60% lower than that of evolocumab [21]. At present, inclisiran is indicated in adults with primary dyslipidemia or mixed dyslipidemia in combination with other lipid-lowering therapies, in patients unable to reach LDL-C goals with the maximum tolerated dose, or alone or in combination with other lipid-lowering therapies in patients who do not tolerate or have contraindications to statins [22]. The ongoing trials (the ORION trial is the biggest one) will probably extend the indications for inclisiran and clarify whether the infrequent administration of inclisiran in monotherapy and/or in combination with statins might further improve the prognosis and outcomes compared with other available lipid-lowering therapies, including PCSK9i.

Furthermore, the 2019 ESC/EAS Guideline for dyslipidemia management recommended that every adult should have at least one Lipoprotein(a) (Lp(a) serum level assessment in their lifetime [2]. Lp(a) is a liver-derived LDL-like particle, with an extra protein called apolipoprotein (a) covalently bound to apolipoprotein B-100 by a single disulfide bond [23]. Previous studies have demonstrated that Lp(a) can promote atherosclerosis, inflammation and thrombosis, and is an independent risk factor for CAD and aortic valve stenosis. Some studies have shown that Lp(a) is associated with an increased risk of CV events irrespective of LDL-C [24], but others have demonstrated that Lp(a) is associated with plaque volume and MACE when LDL-C levels are high, and this association does not exist when LDL-C levels are low [25]. Although Lp(a) is recognized as a risk factor for CAD, a therapeutic strategy is not yet defined. First of all, the effects of statins on plasma Lp(a) concentrations have been inconsistent [26], PCSK9 inhibitors have shown to decrease Lp(a) blood concentration and consequently to reduce the risk of recurrent events following acute coronary syndromes; in particular, alirocumab has shown that each reduction of 5 mg/dL in Lp(a) serum level is associated with a reduction of coronary artery events of 2.5% [27]. The FOURIER trial has documented that patients with higher baseline Lp(a) levels and treated with evolocumab tend to experience greater reduction in major coronary events than patients with lower levels; furthermore, an absolute reduction of 34 nmol/L in Lp(a) blood concentration from baseline is correlated to a reduction of 20% in the relative risk of CV events [16]. The most promising Lp(a)-lowering drug is TQJ230 (Pelacarsen), a hepatocyte-directed antisense oligonucleotide targeting the LPA gene mRNA that reduces Lp(a) by up to 80%. The Lp(a)HORIZON trial has the aim of evaluating the impact of TQJ230 on MACE [28].

In conclusion, the current European Guidelines have further reduced the therapeutic levels of LDL-C in patients at high and very high CV risk in line with the concept “The lower, the earlier, the longer the better”: the greater the absolute reduction in LDL-C, obtained with an early start and long duration of appropriate therapy, the greater the benefit in terms of CV risk reduction. Nevertheless, observational clinical studies have shown a significant gap between the targets recommended by the Guidelines and the LDL-C values obtained in the real world; therefore, the guidelines of the main American and European scientific societies recommend the use of a correct lifestyle and optimal lipid-lowering therapy in all patients who have a documented CV event in history. Currently, the therapeutic strategies of combining multiple lipid-lowering drugs represent the most effective option for achieving the recommended targets. The introduction of new molecules, such as BA and inclisiran, further expands the therapeutic armamentarium available for the management of hypercholesterolemia and, thanks to the infrequent administration regimen of inclisiran, it is expected to increase the number of patients who maintain their therapeutic goals, especially in patients struggling to comply with daily (statins, ezetimibe) or biweekly pharmacotherapy (PCSK9i).
